# The metabolic hypothesis is more likely than the epileptogenic hypothesis to explain stroke-like lesions

**DOI:** 10.12688/wellcomeopenres.15758.2

**Published:** 2020-06-24

**Authors:** Josef Finsterer

**Affiliations:** 1 Krankenanstalt Rudolfstiftung, Messerli Institute, Vienna, 1180, Austria

**Keywords:** mtdna, mitochondrial, stroke-like, epilepsy, stroke-like lesion

## Abstract

Stroke-like episodes (SLEs) are a hallmark of mitochondrial encephalopathy, lactic acidosis, and stroke-like episode (MELAS) syndrome but occur in other mitochondrial disorders (MIDs) as well. The morphological equivalent of the SLE is the stroke-like lesion (SLL) on magnetic resonance imaging (MRI). The pathophysiology of SLLs is under debate, but several hypotheses have been raised to explain the phenomenon. Of these, the metabolic, epileptogenic, and vascular hypotheses are the most frequently discussed. There are several arguments for and against these hypotheses, but a consensus has not been reached which of them provides the correct explanation. A recent consensus statement generated by a panel of experts applying the Delphi method, favoured the epileptogenic hypothesis and recommended treatment of SLEs with antiepileptic drugs, irrespective if the patient presented with a seizure or epileptiform discharges on electroencephalography (EEG) or not. We disagree with this general procedure and provide the following arguments against the epileptogenic hypothesis: 1. not each SLE is associated with seizures. 2. epileptiform discharges may be absent on EEG during a SLE. 3. SLLs are not restricted to the cortex. 4. antiseizure-drugs (ASDs) may not prevent the progression or recurrence of a SLL. 5. ASDs may terminate seizures but no other phenotypic feature of a SLE. 6. patients already under ASDs are not immune from developing a SLL. 7. SLLs usually last longer than seizures. 8. no animal model supports the epileptogenic hypothesis. The strongest arguments for the metabolic hypothesis are that SLLs are not confined to a vascular territory, that the oxygen-extraction fraction within a SLL is reduced, and that there is hypometabolism within a SLL on FDG-PET. SLLs may respond to antioxidants, NO-precursors, steroids, or the ketogenic diet. ASDs should be applied only if there is clinical or electrophysiological evidence of seizure-activity.

## Correspondence

We read with interest the consensus statement by Ng *et al.* about the pathogenesis and treatment of stroke-like lesions (SLLs) ^1^. We disagree with the consensus paper in the point that it does not consider arguments for alternative pathomechanisms explaining the development of a SLL.

There are several arguments against the epileptogenic hypothesis. First, not all patients with a mitochondrial disorder (MID) who ever experienced a SLL also have an individual history positive for epilepsy ^2^. Thus, the statement that “seizures are commonly present at the onset of stroke-like episodes (SLEs)” is not comprehensible ^1^. Second, the electroencephalography (EEG) during a SLL does not reveal epileptogenic activity in most cases, irrespective if the patient experienced a seizure or not ^3^. Third, SLLs are not restricted to the cortex ^4^. Though SLLs originate from stressed cortical layers in the majority of cases, there are also extra-cortical locations of SLLs. SLLs have been reported in the thalamus, midbrain, pons, and the cerebellum ^5^. There are even indications that SLLs may develop within the optic nerve ^6^. Fourth, anti-seizure drugs (ASDs) may not exhibit a beneficial effect on the dynamics, development, and outcome of a SLL ^7^. ASDs may stop seizure activity but may not affect morphology, extension, or dynamics of a SLL or its other clinical manifestations. Fifth, patients with SLLs may have seizures timely-unrelated to the occurrence of a SLL ^8^. Sixth, patients already under ASDs for previous seizures may not be saved from developing a SLL nonetheless. Even if ASDs are given for a SLL ^9^, this may not prevent the development of a second or third SLL in the same or another location. Seventh, a SLL can last for months, whereas a seizure is usually a limited event unless it is an epileptic state ^10^. Eighth, there is no animal model of a MID available in which triggering of seizures induces the development of a SLL.

More plausible than the epileptogenic hypothesis to explain the appearance of a SLL is the metabolic hypothesis ^11, 12^. There are several arguments in favour of the metabolic hypothesis as a pathogenetic model to explain the occurrence of a SLL. First, a MID is a metabolic disorder with a defect in the mitochondrial energy production ^11^. Functionally impaired mitochondria may not be resistant against increased oxidative stress resulting in a metabolic breakdown, cellular dysfunction, and finally degeneration or apoptosis of neurons, glial cells, endothelial cells, vascular smooth muscle cells, or pericytes. Increased oxidative stress may be due to increased physical or psychological requirements, infections, cerebral ischemia, seizure activity, intoxication, or increased metabolic demand ^13, 14^. Second, oxygen-extraction within the SLL is reduced on oxygen-extraction fraction (OEF)-MRI ^15, 16^ suggesting that impaired mitochondria can no longer utilise oxygen properly. As with increased oxygen concentrations in venous blood from MID patients, cells within the SLL are no longer capable of utilising oxygen sufficiently. They most likely change their energy metabolism to anaerobic glycolysis or produce ATP within the cytoplasm by means of glycolysis. Third, in the early stages of a SLL, alterations are predominantly found in cortical areas with particularly high oxidative stress ^4^. In accordance with the frequent location of a SLL in the occipito-temporal regions, one of the highest metabolic demands has been found in the occipital cortex ^17^. This is probably attributable to the density of neurons, which is the highest in the occipital cortex ^17^. Furthermore, neurons from the visual cortex are exposed to a higher glutaminergic input from dendrites compared to the motor cortex ^4^ with a high demand to maintain ionic homeostasis after excitatory depolarisation ^18, 19^. Fourth, dendrite-rich cortical areas are particularly vulnerable to hypoxia ^20^ and the density of mitochondria is particularly high in dendrites ^21^. Fifth, serum amino acids may be decreased at onset of a SLE to increase shortly afterwards again ^22^. Low levels of serum amino acids suggest that energy during the focal, cerebral, metabolic crisis is generated by utilisation of amino acids. Sixth, increased lactate peaks and decreased N-acetylaspartate (NAA)-peaks in m.3243A>G carriers on MR-spectroscopy ^23, 24^ can be reversed by intravenous L-arginine ^25^. Generally, the NO-precursor L-arginine seems to exhibit a beneficial effect on the extension, progression, duration, and outcome of a SLL, why it has been approved by the FDA as a treatment of SLLs.

In summary, we agree that seizures may occasionally trigger the development of a SLL but we disagree that this is the general pathophysiology. Other triggers should, as outlined above, be considered as well. SLLs may develop in response to stress as neurons carrying mutated mitochondria are no longer capable to meet an increased metabolic demand. Extra-cortical SLLs are no argument against the metabolic hypothesis as high energy demand may not only occur in the cortex but also in other cerebral locations, depending on the current tasks of a network or circuit. High amounts of sensory input may, for example, stress thalamic or cerebellar neurons leading to a SLL there. Understanding the pathophysiology of SLLs is a prerequisite to optimally manage them.

## Data availability

### Underlying data

No data are associated with this article
