# Supplementary material for: The metabolic hypothesis is more likely than the epileptogenic hypothesis to explain stroke-like lesions
Source: Wellcome Open Res. 2020 Jun 24;5:51. Originally published 2020 Mar 26. [Version 2] doi: 10.12688/wellcomeopenres.15758.2 (PMC7324943; doi:10.12688/wellcomeopenres.15758.2)
Supplement: Peer review history

# Peer review: Reviewer response for version 2

**DOI:** 10.21956/wellcomeopenres.17664.r39228

**Published:** 2020-06-29

**Authors:** Mirian C.H. Janssen

**Affiliations:** 1 Amalia Children’s Hospital, Department of Pediatrics, Radboud Center for Mitochondrial Medicine, Radboud University Medical Center, Nijmegen, The Netherlands

No new additional comments

Are arguments sufficiently supported by evidence from the published literature or by new data and results?

No

Is the conclusion balanced and justified on the basis of the presented arguments?

No

Is the rationale for commenting on the previous publication clearly described?

Yes

Are any opinions stated well-argued, clear and cogent?

No

Reviewer Expertise:

Metabolic disease in adults, MItochondrial disease, MELAS

I confirm that I have read this submission and believe that I have an appropriate level of expertise to confirm that it is of an acceptable scientific standard.

# Peer review: Reviewer response for version 1

**DOI:** 10.21956/wellcomeopenres.17283.r38962

**Published:** 2020-06-19

**Authors:** Mirian C.H. Janssen

**Affiliations:** 1 Amalia Children’s Hospital, Department of Pediatrics, Radboud Center for Mitochondrial Medicine, Radboud University Medical Center, Nijmegen, The Netherlands

The author comments on the recently published European consensus on management of stroke like episodes.

 The goal of the consensus was not to elaborate extensively on the pathomechanism of SLLs. In the introduction it is described that underlying mechanism of stroke-like episodes remains unclear. The vascular theory is explained and also the theory of neuronal hyper-excitability. It is emphasized that there are controversies surrounding the mechanisms. The recommendations are derived from the consensus approach, based on clinical experience from the leading mitochondrial clinical services of several European countries and the appraisal of current literature. They acknowledge that the majority of the recommendations are not evidence-based due to the inherent challenges of conducting clinical trials in rare diseases.

 The author states that the metabolic hypothesis is more likely to explain SLL than the epileptogenic hypothesis and puts several arguments against the epileptic theory like “patients with SLLs may have seizures not time-related to the occurrence of a SLL’’, “patients already under ASDs for previous seizures are not be saved from developing a SLL nonetheless, even if ASDs are given for a SLL’’ and there is no animal model of a MID available in which triggering of seizures induces the development of a SLL.

 These arguments are not convincing against the epileptic theory nor pro the metabolic theory. The argument “antioxidants and cofactors can be beneficial in some patients with a SLL a well as steroids which may re-establish the blood-brain barrier disrupted by the metabolic defect’’, has not been proven at all. These are suggestions which have not been proven in a proper clinical trial. That is also the case for the ketogenic diet.

 Although the metabolic theory sounds plausible, the author has no valid data to substantiate this theory, it is only based on anecdotal case reports. For this reason I find the article not suitable for indexing. A more proper critical appraisal on evidence concerning the metabolic theory would be valuable.

Are arguments sufficiently supported by evidence from the published literature or by new data and results?

No

Is the conclusion balanced and justified on the basis of the presented arguments?

No

Is the rationale for commenting on the previous publication clearly described?

Yes

Are any opinions stated well-argued, clear and cogent?

No

Reviewer Expertise:

Metabolic disease in adults, MItochondrial disease, MELAS.

I confirm that I have read this submission and believe that I have an appropriate level of expertise to state that I do not consider it to be of an acceptable scientific standard, for reasons outlined above.

# Response

**Published:** 2020-06-19

**Authors:** josef finsterer

**Affiliations:** krankenanstalt rudolfstiftung, Austria

**The author comments on the recently published European consensus on management of stroke like episodes. The goal of the consensus was not to elaborate extensively on the pathomechanism of SLLs. In the introduction it is described that underlying mechanism of stroke-like episodes remains unclear. The vascular theory is explained and also the theory of neuronal hyper-excitability. It is emphasized that there are controversies surrounding the mechanisms. The recommendations are derived from the consensus approach, based on clinical experience from the leading mitochondrial clinical services of several European countries and the appraisal of current literature. They acknowledge that the majority of the recommendations are not evidence-based due to the inherent challenges of conducting clinical trials in rare diseases.**

If the recommendations of the consensus group are not evidence-based, as indicated by Prof. Janssen, they have a similar strength as other expert opinions.

**The author states that the metabolic hypothesis is more likely to explain SLL than the epileptogenic hypothesis and puts several arguments against the epileptic theory like “patients with SLLs may have seizures not time-related to the occurrence of a SLL’’, “patients already under ASDs for previous seizures are not be saved from developing a SLL nonetheless, even if ASDs are given for a SLL’’ and there is no animal model of a MID available in which triggering of seizures induces the development of a SLL. These arguments are not convincing against the epileptic theory nor pro the metabolic theory.**

These and other arguments were not raised in favour of the metabolic hypothesis but against the epileptogenic hypothesis. If the consensus group favours antiepileptic drug (AED) treatment for any SLL, irrespective if there are seizures or epileptiform discharges on EEG, where is the rationale for AEDs?    

**The argument “antioxidants and cofactors can be beneficial in some patients with a SLL a well as steroids which may re-establish the blood-brain barrier disrupted by the metabolic defect’’, has not been proven at all. These are suggestions which have not been proven in a proper clinical trial. That is also the case for the ketogenic diet.**

We agree, that the evidence for antioxidants, steroids, and the ketogenic diet (KD) is low. Only a few clinical and experimental studies are available that gave promising results with these compounds [Frey et al. Biochim Biophys Acta Mol Basis Dis 2017, Steriade et al. Pediatr Neurol 2014, Haefeli et al, PlosOne 2011]. However, NO-precursors (eg. L-arginine), and not antioxidants, steroids, and the KD are the most widely proposed treatment for SLLs. For supporting NO-precursors, controlled studies are available [Rodan et al. PlosOne 2015, Koga et al. J Neurol 2018, El-Hattab et al, Mol Genet Metab 2016].

**Although the metabolic theory sounds plausible, the author has no valid data to substantiate this theory, it is only based on anecdotal case reports. For this reason I find the article not suitable for indexing. A more proper critical appraisal on evidence concerning the metabolic theory would be valuable.**

We do not agree that only case reports are available for arguing in favour of the metabolic hypothesis. There are  a few case-control, observational, and prospective clinical trials [Rodan et al. PlosOne 2015, Koga et al. J Neurol 2018, El-Hattab et al, Mol Genet Metab 2016, Sudo et al. Brain Development 2014] and experimental trials [Desquiret-Demas et al. Biochim Biophys Acta 2012] available. Furthermore, case reports do not mean no evidence, just lower evidence than randomised, controlled studies. Since no very high level studies are currently available, a critical appraisal of the metabolic theory can rely only on the currently available data and these are the few observational, controlled studies, case reports, and expert opinions.

# Peer review: Reviewer response for version 1

**DOI:** 10.21956/wellcomeopenres.17283.r38255

**Published:** 2020-04-14

**Authors:** Claude Steriade

**Affiliations:** 1 Epilepsy Center, New York University Langone Health, New York City, NY, USA

Finsterer provides an alternative proposal to the recently published consensus statement on diagnosis and management of stroke-like episodes related to mitochondrial disorders, in which seizures are highlighted as a core aspect of stroke-like episodes which should be targeted through therapeutics. The author outlines several lines of evidence pointing to an alternative metabolic hypothesis, in which seizures can be a consequence but are not the root cause of neuronal injury. The arguments are convincing and provide a framework for a more rational and well rounded pathophysiology of stroke like episodes, and provides the basis for possible changes to the published consensus statement.

Are arguments sufficiently supported by evidence from the published literature or by new data and results?

Yes

Is the conclusion balanced and justified on the basis of the presented arguments?

Yes

Is the rationale for commenting on the previous publication clearly described?

Yes

Are any opinions stated well-argued, clear and cogent?

Yes

Reviewer Expertise:

Epilepsy

I confirm that I have read this submission and believe that I have an appropriate level of expertise to confirm that it is of an acceptable scientific standard.

# Response

**Published:** 2020-04-18

**Authors:** josef finsterer

**Affiliations:** krankenanstalt rudolfstiftung, Austria

Thank you for these valuable comments
